# Medical Society of the County of Erie. Memorial Meeting

**Published:** 1903-04

**Authors:** Franklin C. Gram


### SOCIETY PROCEEDINGS.

### Medical Society of the County of Erie.

### Memorial Meeting.

HERMAN MYNTER, M. D.

REPORTED BY FRANKLIN C. GRAM, M. D., Secretary.

THE Medical Society of the County of Erie met in special session February 11, 1903, in the parlors of the Y. M. C. A., Buffalo, in response to a call by President ERNEST WENDE, who stated that he had requested the presence of the members for the purpose of taking appropriate action on the death of Dr. Herman Mynter, an honored member of this society.

Dr. HENRY REED HOPKINS moved that the president appoint a committee of five to submit a suitable memorial. The resolution was adopted and the president appointed Drs. H. R. Hopkins, Charles G. Stockton, Joseph Fowler, Eugene A. Smith and James W. Putnam.

The committee presented the following, which was adopted:

Forasmuch as it hath pleased Almighty God, in His wise providence, to take out of the world the soul of our deceased brother, Dr. Herman Mynter, we, the Medical Society of the County of Erie, therefore declare and pronounce this estimate and testimonial of his life, work and character.

Herman Mynter was generously endowed by an honorable ancestry, with rare gifts of mind and person. These gifts, fortunately, had been well developed by his educational training, the best the schools and universities of Europe could give; and when in the days of his early manhood he came to Buffalo to become a loyal and enthusiastic American citizen, and a member of its medical profession, he came not empty handed, but magnificently equipped; not needing care, but ready and able to care for others. During his entire life, as strength and health permitted, he practised his profession, which he ever honored and adorned, with devotion to its highest ideals, and with distinguished success.

Dr. Mynter was a frequent contributor to the literature of medicine, and his contributions ever bore the mark of distinct individuality, unusual mastery of its scientific principles and progress-making originality. His success as a teacher was pronounced, because he ever gave to this portion of his work—as to all his efforts,—his very best. The monument of his life, more lasting and beautiful than carven granite, or sculptured marble, is his practice—where thousands of human lives have been blest by his intelligent conception of scientific principles and his skilful application of these principles for the relief of suffering, deformity and disease.

We loved him and we will cherish his memory, not only for his brilliant professional achievements of mind and hand, but moreover for his record of clean, manly and blameless walk and conversation, the proof of the intrinsic nobility of his character. Therefore

*Resolved*, That this minute be accepted, inscribed upon our records, and a copy transmitted to the family of our deceased brother.

HENRY R. HOPKINS,
CHARLES G. STOCKTON,
JOSEPH FOWLER,
JAMES W. PUTNAM,
EUGENE A. SMITH.

DR. EUGENE A. SMITH said he had been closely associated with Dr. Mynter for ten years, as his assistant in the Sisters of Charity Hospital, the Niagara Medical College and in his private practice. He spoke of his professional and other characteristics, his phenomenal success as a surgeon, his ability as a teacher of surgery, as a clinician, and his diagnostic intuition.

DR. JAMES W. PUTNAM appreciated his ability. He was a leader wherever he went, and the honesty of his convictions was always apparent. Among members of his own profession he was always frank, and acknowledged no man as his superior. He achieved fame and established himself as a careful and thorough surgeon during the first year of his practice in Buffalo. In spite of adversity, which would have subdued a weaker man, he held his place in the surgical ranks to the end.

DR. IRVING M. SNOW spoke of Dr. Mynter's ancestors, who were famous in the history of their country.

DR. JAMES S. SMITH had known Dr. Mynter since he came to Buffalo, and believed himself better for such acquaintance. He mentioned some of Dr. Mynter's first operations for appendicitis, on which he later became an authority. He was a great student as well as a great surgeon.

DR. EUGENE WASDIN, of the United States Marine Hospital Service, added a personal tribute, in which he mentioned his association with Dr. Mynter in the culminating case of his life, during the trying time following the assassination of President McKinley.

Dr. Charles G. Stockton joined with other members in the expression of grief. Dr. Mynter occupied a unique place in the community. He combined a childlike simplicity with manly strength. He put great force and intensity of feeling into every question, but at the end of every controversy Mynter was himself again and never bore a grudge.

Dr. J. B. Coakley likewise emphasised the simplicity of Dr. Mynter's life and related some personal incidents.

Dr. P. W. Van Peyma dwelt upon his honesty, brusqueness, frankness and generosity, which were always in evidence.

At the conclusion of these remarks the society adjourned.

